# The Effect of Chia Seeds on High-Density Lipoprotein (HDL) Cholesterol

**DOI:** 10.7759/cureus.40360

**Published:** 2023-06-13

**Authors:** Brian Dickens, Mana Sassanpour, Evan L Bischoff

**Affiliations:** 1 Family Medicine, Edward Via College of Osteopathic Medicine, Blacksburg, USA; 2 Family Medicine, Primary Medical Group, Ventura, USA

**Keywords:** hdl cholesterol, lipid-lowering, chia seeds, dietary modification, triglyceride, ldl cholesterol

## Abstract

Context: Chia seeds are touted as a healthy food capable of providing a beneficial effect on high-density lipoprotein (HDL) cholesterol. Similar claims have been made for oats in various forms, claiming that they improve cholesterol or are in some way “heart healthy.”

Objective: This study aims to demonstrate the effect of daily consumption of chia seeds, if any, on HDL cholesterol levels and compare this to the effects of oats on HDL levels.

Methods: This pilot study is a randomized controlled trial performed at an academic primary care center. Participation was voluntary, and all participants provided written consent prior to enrollment. There were no exclusion criteria other than that participants must be adults and willing to come in to get their cholesterol profiles checked at the beginning and end of the study. The participants consumed their assigned breakfast in a standard serving size for a month with blood draws and weights recorded before and after the diet. The patients’ cholesterol profiles were also compared with their weights. To standardize the delivery of chia seeds, the group consuming chia seeds mixed them with oatmeal. The three meal groups consisted of Cheerios (red group), oatmeal (white group), and oatmeal with chia seeds (blue group). Initially, there were a total of 11 subjects, three in the red group, four in the white group, and four in the blue group. Two subjects were lost to follow-up, one each from the red and white groups. Statistical analysis including one-way analysis was done with the means, Wilcoxon/Kruskal-Wallis, and one-way analysis tests.

Results: The results showed a similar weight gain pattern between the three groups, with an average of 2.8 lbs gained in the red group, 2.4 lbs gained in the white group, and 2.6 lbs gained in the blue group. The average HDL levels decreased by 1.00 mg/dL in the red group (standard deviation (SD) 2.82843), increased by 2.00 mg/dL in the white group (SD 8.88819), and increased by 5.75 mg/dL in the blue group (SD 8.01561). The HDL:total cholesterol ratios decreased by 0.00748 in the red group (SD 0.002660), an average of 0.04053 in the white group (SD 0.028138), and an average of 0.01614 in the blue group (SD 0.023808).

Conclusion: The results suggest that both chia seeds and oatmeal may be good dietary sources to naturally increase HDL cholesterol, more substantially so with the diet including chia seeds, but may or may not improve total cholesterol:HDL ratios. The effect of weight gain is unclear, as all groups gained weight similarly. Hence, further study is warranted.

## Introduction

Multiple avenues for improving lipid levels and cardioprotective factors have been explored both medically and nutritionally. Pills may sometimes help your health, but many people are just tired of eating one pill after another; they are often expensive and full of side effects [1]. They also have their limits. Statin medications, in particular, do a great job of lowering low-density lipoprotein (LDL) cholesterol in most people, but they tend to have a less impressive effect on high-density lipoprotein (HDL) [2]. Higher HDL levels are widely considered cardioprotective [3]. Foods capable of naturally increasing HDL cholesterol may have cardiovascular benefits [4]. Chia seeds are an interesting category; because they are a staple grain in many cultures, their potential health benefits are well documented. Chia seeds are touted as a healthy food with a beneficial effect on HDL cholesterol [5]. Similar claims have been made for oats, claiming they improve cholesterol or are in some way “heart healthy.” A meta-analysis on the cardioprotective effects of oats found that while the LDL levels were improved, the HDL levels did not change [6]. Another study by Maki et al. in the *Journal of the American Dietetic Association *showed a reduction in total and LDL cholesterol, but no change in HDL cholesterol was found between the control group and subjects given oats as a part of a weight reduction diet [7].

A study in *Poultry Science* by Ayerza and Coates showed increased omega-3 fatty acid content in the yolks of eggs from hens fed with chia seeds versus a standard diet [8]. The protective effect of omega-3s is well documented both as a way to decrease lipid levels and to add cardioprotective effects. Chia seeds have the potential to improve cholesterol levels with their high omega-3 content. Another potential benefit to cardiovascular numbers could be chia seed’s high fiber content [9].

Chia seeds, when combined with oats, soy, and nopal (a type of cactus), were shown to reduce triglyceride levels in a study by Guevara-Cruz et al. [10]. Chia seeds are widely consumed in Latin America and associated with a favorable lipid profile due to their high omega-3 content, but scientific evaluation of their safety and efficacy as a medicinal food is lacking [11].

Chia seeds were also shown to reduce the LDL levels in obese rat models. This study also noted a decrease in postprandial glucose [12]. This effect, along with a decrease in the waist circumference, was also observed in another study investigating the total amount of triglycerides in the blood of rats after consuming a chia seed diet [13]. Chia seed extract was also seen to decrease triglyceride levels in *Caenorhabditis elegans *models [14]. Hence, the potential of chia seeds includes reducing some of the health issues related to obesity.

Chia seeds may hold potential as a dietary intervention to raise HDL cholesterol in the American breakfast diet. This pilot study aims to evaluate the effect of one dietary modification with a typical implementation as an individual would reasonably do when making a lifestyle modification intended to be maintained long term. As such, rather than rigid dietary restrictions, subjects were given one prescribed breakfast and were otherwise free to eat a typical calorie-unrestricted diet during the study.

## Materials and methods

This pilot study is a small, randomized controlled trial conducted at an academic primary care center (Academic Primary Care Associates, Blacksburg, Virgina, USA). At the beginning of the study, each participant was randomly assigned to the red, white, or blue group. They were then assigned a number within that group. The red group was instructed to consume one serving of Cheerios according to package instructions each morning for 30 days, the white group with one packet of instant oatmeal, and the blue group with one packet of instant oatmeal with two tablespoons of chia seeds.

The rationale for combining the chia seeds with oatmeal in the blue group was twofold. First, consuming dry chia seeds alone presented a potential choking hazard. Second, by combining the chia seeds with oatmeal, already being consumed by one group in the study, the variability of absorption and effects of the second substance being consumed with it could be standardized.

The participants consumed their assigned breakfast in a standard serving size for a month with blood draws and weights recorded before and after the diet. The patients’ blood was taken twice with intent to measure the cholesterol profile, including total triglycerides, LDL cholesterol, and HDL cholesterol. Initially, there was a total of 11 subjects, three in the red group, four in the white group, and four in the blue group. Two subjects were lost to follow-up, one each from the red and white groups, respectively.

Statistical analysis including one-way ANOVA was performed with the means, Wilcoxon/Kruskal-Wallis, and one-way analysis tests. The statistical analysis was hampered by the small sample size.

This pilot study was approved by the Edward via College of Osteopathic Medicine Institutional Review Board, Blacksburg, Virginia, United States (approval number 2015-011).

## Results

**Figure 1:**
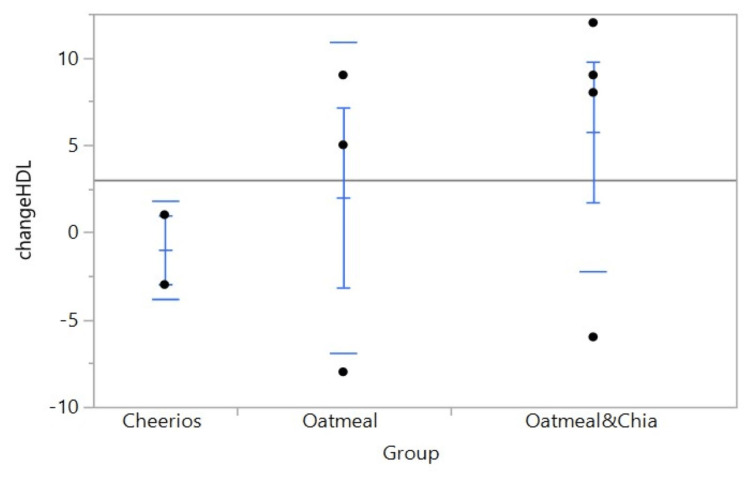
One-way analysis of the percent change of HDL by group HDL: high-density lipoprotein

**Figure 2:**
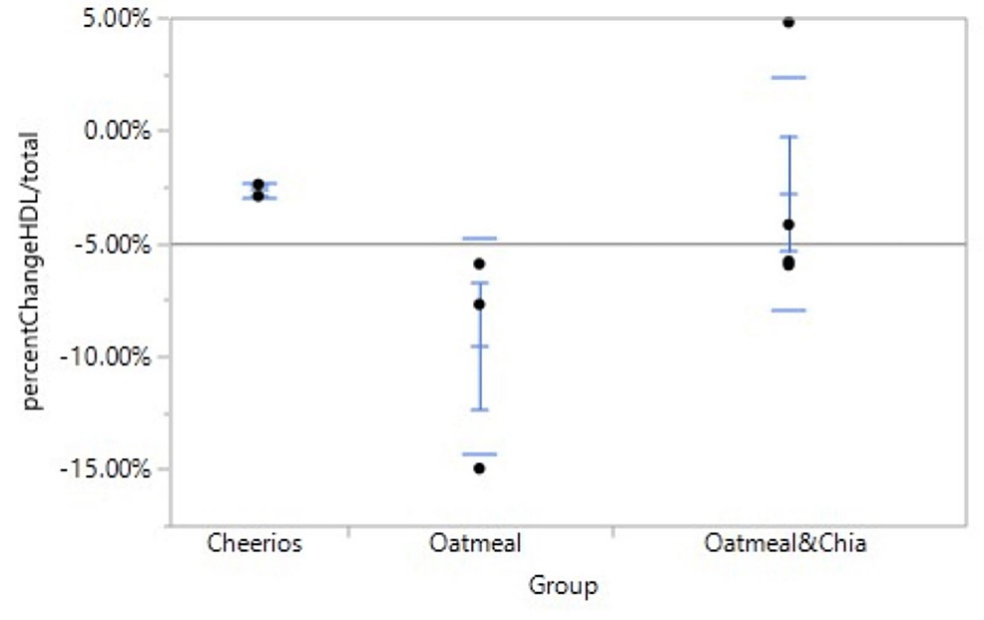
One-way analysis of the percent change of HDL/total cholesterol by group HDL: high-density lipoprotein

Our first experiment was to determine if consuming chia seeds affected the overall weight by the end of the study. The results showed a similar weight gain pattern between the three groups, with an average of 2.8 lbs gained in the red group, 2.4 lbs gained in the white group and 2.6 lbs gained in the blue group. In our next experiment, we aimed to find any relation between chia seed consumption and HDL levels. The average HDL levels decreased by 1.00 mg/dL in the red group (standard deviation (SD) 2.82843), increased by 2.00 mg/dL in the white group (SD 8.88819), and increased by 5.75 mg/dL in the blue group (SD 8.01561) (Figure 1, Table 1). Finally, we aimed to determine if the overall ratio of HDL to total cholesterol was changed with chia seed consumption. The HDL:total cholesterol ratios decreased by 0.00748 in the red group (SD 0.002660), an average of 0.04053 in the white group (SD 0.028138), and an average of 0.01614 in the blue group (SD 0.023808) (Figure 2, Table 2, Table 3).

**Table 1:** Means and standard deviations of the HDL changes between groups HDL: high-density lipoprotein; Std Dev: standard deviation; Std Err Mean: standard error mean

Level	Number	Mean	Std Dev	Std Err Mean	Lower 95%	Upper 95%
Cheerios	2	-1	2.82843	2	-26.41	24.412
Oatmeal	3	2	8.88819	5.1316	-20.08	24.079
Oatmeal and Chia	4	5.75	8.01561	4.0078	-7	18.505

**Table 2:** Wilcoxon/Kruskal-Wallis tests (rank sums) of the HDL:total cholesterol percent changes HDL: high-density lipoprotein

Level	Count	Score Sum	Expected Score	Score Mean	(Mean-Mean0)/Std0
Cheerios	2	7	10	3.5	-0.735
Oatmeal	3	13.5	15	4.5	-0.259
Oatmeal and Chia	4	24.5	20	6.125	0.984

**Table 3:** One-way test and chi-square approximation of the HDL:total cholesterol percent changes HDL: high-density lipoprotein; DF: degree of freedom; Prob>ChiSq: probability>chi-square

Chi-square	DF	Prob>ChiSq
1.3866	2	0.4999

## Discussion

The results suggest that both chia seeds and oatmeal may be good dietary sources to naturally increase HDL cholesterol, but they may or may not improve total HDL:cholesterol ratios. The effect of weight gain is unclear, as all groups gained weight similarly. Whether this was due to a carbohydrate-heavy breakfast or chance is uncertain because of the small sample size and lack of a control group without a standardized breakfast. The results of the study indicate potential benefits to patients and demonstrate the need for further study.

The more significant increase in HDL in the blue group could be due to a synergistic effect between both oatmeal and chia seeds. It is also possible that the chia seeds alone were the primary factor in the increased HDL. To test the effects of chia seeds alone, it would have been possible for the chia seed group to simply hydrate the chia seeds in water and eat the resulting chia gel. However, the study team believed that requiring such an unpalatable breakfast for 30 days would likely lead to a high attrition rate in an already small pilot study.

Since this study concluded, a few studies have come out with updated information regarding the cardioprotective effects of chia seeds. In rats, it was found that chia seeds improve cardiac antioxidant defenses. They help restore the cardiac unbalanced redox state of dyslipidemic insulin-resistant rats. Chia seeds also reduce left ventricle collagen deposition and dyslipidemia in rat models. Hence, their consumption could be a beneficial recommendation for future dietary concerns of patients with diabetes and is worth exploring in subsequent studies [15].

Further testing should also be done to determine if dosing can be optimized to produce the best possible results. Two tablespoons of chia seeds is a relatively small dose, so a higher intake may well do more. The reason to start the dosing slow is that chia seeds are high in oxalates and most cases of nephrolithiasis are the result of calcium oxalate stones. Although none of the participants had a history of nephrolithiasis and none reported a development of symptomatic nephrolithiasis during or immediately after the study, it was a theoretical concern with higher doses. Thus, the daily dose in this study was limited to two tablespoons.

Another factor potentially affecting the outcome was the lack of stricter control with other meals and activities. The study was designed to optimize the clinical utility of the findings of a single dietary change rather than to strictly isolate one nutritional component, so subjects were instructed to eat, exercise, and live their lives as they have prior to the study, with the exception of having one meal per day for 30 days.

Theoretically, any of the groups could have eaten their prescribed breakfast and immediately followed it with a high-fat or high-cholesterol meal. Thus, the results reflect one dietary habit change along with any downstream effects, such as increased or decreased hunger and increase or decrease in calories consumed throughout the rest of the day. A calorie limit or target could have been implemented, but doing so may have skewed the full effects of the change seen with typical daily life.

The dietary use of chia seeds also has the potential to help obese patients trying to improve cholesterol profiles. A mice model study found that chia seeds help bring down LDL cholesterol in obese mice [12]. As this pilot project did not assess for obesity, this could be a future experimental group to include. LDL levels before and after consuming chia seeds could be compared in obese patients.

The biggest limitation of this study is the sample size. As this is a pilot study, patterns can be appreciated, but caution must be taken when drawing conclusions. Further testing is warranted for cardiac outcomes. The results do not necessarily correlate to a reduction in cardiovascular endpoints, such as myocardial infarction, cerebrovascular accident, or death. Nonetheless, the suggestion that the addition of something as simple as chia seeds to a diet may increase HDL cholesterol substantially is intriguing. Hence, a larger scale study is warranted.

## Conclusions

The results suggest that chia seeds and oatmeal may be good dietary sources to naturally increase HDL cholesterol, more substantially so with a diet including chia seeds, but they may or may not improve total cholesterol:HDL ratios. The effect on weight gain is unclear. The limiting factors of this study include the small sample size, separation of effects of oatmeal and chia seeds, and dose of chia seeds consumed. Although data suggest that there may be some beneficial effects, further study is still warranted.
